# Improvement in the identification and management of inadvertent hypothermia in the critically ill: an audit cycle

**DOI:** 10.1186/cc13194

**Published:** 2014-03-17

**Authors:** J Barnes, R Darke, A Irving, S Wright

**Affiliations:** 1 Freeman Hospital, Newcastle upon Tyne, UK

## Introduction

The purpose of this study was to assess our practice in identifying and managing inadvertent hypothermia and whether this could be improved by education and introduction of a protocol. Hypothermia is associated with multiple physiological abnormalities including reduced myocardial contractility, peripheral vasoconstriction, increased infection risk and impaired coagulation [1]. Inadvertent hypothermia may therefore be an avoidable risk factor in the critically ill. The UK National Institute of Clinical Excellence has issued guidance for avoidance of inadvertent hypothermia (temperature <36°C) during the perioperative period. We audited our practice against three standards from these guidelines: all patients should have at least 4-hourly temperature observations; no patient should become inadvertently hypothermic; and all inadvertently hypothermic patients should be rewarmed.

## Methods

Data were collected prospectively. Body temperature was recorded routinely by nursing staff using a tympanic thermometer. We noted any occasion where the body temperature dropped below 36°C along with any associated interventions - such as the use of additional bed sheets or a forced air warming device. After the first audit period a simple education programme was delivered. We also introduced a departmental protocol for the prevention and management of inadvertent hypothermia. Six months later we re-audited our practice.

## Results

Data were collected from 130 patients (2,931 patient-hours) in the first audit period and from 87 patients (2,070 patient-hours) in the second audit period. In the first period 29% of patients had at least 4-hourly temperature measurements compared with 40% in the second period (*P *< 0.01). The average number of overdue temperature observations per day was 1.4 in the first period and 0.9 in the second (*P *< 0.01). Twenty-four per cent of patients became hypothermic in the first period compared with 22% in the second (*P *= 0.07); however, the time these patients remained hypothermic reduced from an average of 7.9 hours to 6.1 hours (*P *< 0.01). An intervention was made and documented in 15% of cases in the first period and 46% in the second (*P *< 0.001).

## Conclusion

We saw some improvement following an education programme and introduction of a clinical protocol although there remains room for further improvement.
